# Supplementary material for: Active case-finding policy development, implementation and scale-up in high-burden countries: A mixed-methods survey with National Tuberculosis Programme managers and document review
Source: PLoS One. 2020 Oct 28;15(10):e0240696. doi: 10.1371/journal.pone.0240696 (PMC7592767; doi:10.1371/journal.pone.0240696)
Supplement: S2 Appendix — (DOCX) [file pone.0240696.s002.docx]

**Systematic screening and active case finding (ACF) for early detection of active tuberculosis (TB) – towards a better understanding of national ACF policy development and implementation**

**1 Personal background**

- 1. **Sex**

| Female | Male |
| --- | --- |

- 1. **Age group (years)**

| 20-29 | 30-39 | 40-49 | 50-59 | 60-69 | >70 |
| --- | --- | --- | --- | --- | --- |

- 1. **Country**

|  |
| --- |

- 1. **Organization**

|  |
| --- |

- 1. **Job title**

|  |
| --- |

- 1. **Role**

| Policy-maker | Researcher | Other |
| --- | --- | --- |

*1.6.1 If you identified yourself as a policy-maker or other stakeholder, please indicate if you have training and/or extensive experience as a researcher.*

| Yes | No |
| --- | --- |

*1.6.2 If you identified yourself as a researcher, please indicate if you have training and/or extensive experience as a policy-maker.*

| Yes | No |
| --- | --- |

**1.7 What year did you start working in this job?**

|  |
| --- |

- 1. **What year did you start working in the area of TB?**

|  |
| --- |

**2 ACF policies – general view**

***“Systematic screening for active TB is defined as the systematic identification of people with suspected active TB, in a predetermined target group, using tests, examinations or other procedures that can be applied rapidly.”*** – World Health Organization

**2.1 Specify if you agree with the following statements on the benefits of ACF:** *2.1.1 ACF leads to early detection, diagnosis and treatment.*

| Strongly  agree | Agree | Neither agree nor disagree | Disagree | Strongly disagree |
| --- | --- | --- | --- | --- |

*2.1.2 ACF leads to reduced transmission and incidence of TB.*

| Strongly  agree | Agree | Neither agree nor disagree | Disagree | Strongly disagree |
| --- | --- | --- | --- | --- |

*2.1.3 ACF leads to improved treatment outcomes.*

| Strongly  agree | Agree | Neither agree nor disagree | Disagree | Strongly disagree |
| --- | --- | --- | --- | --- |

*2.1.4 ACF leads to reduced future health system cost.*

| Strongly  agree | Agree | Neither agree nor disagree | Disagree | Strongly disagree |
| --- | --- | --- | --- | --- |

*2.1.5 ACF has positive social and economic consequences for the TB patient.*

| Strongly  agree | Agree | Neither agree nor disagree | Disagree | Strongly disagree |
| --- | --- | --- | --- | --- |

**2.2 Are there other benefits of ACF you would like to mention? Please elaborate.**

|  |
| --- |

**2.3 Why did you agree/disagree with the benefits of ACF? Please elaborate.**

|  |
| --- |

**2.4 How would you rank the importance of ACF among other TB interventions for early case detection? Please rank from 1-7 (1 = most important)**

- Implementing ACF
- Improving knowledge about TB among patients and the community
- Reducing access barriers to health care
- Reducing stigma
- Implementing proven diagnostic tools
- Training health workers
- Other (please specify) _____________________

*2.4.1 Please elaborate on why you ranked the TB interventions for early case detection as you did.*

|  |
| --- |

*2.4.2 ACF contributes to the goals of you National Strategic Plan.*

| Strongly agree | Agree | Neither agree nor disagree | Disagree | Strongly disagree |
| --- | --- | --- | --- | --- |

Please elaborate:

|  |
| --- |

**2.5 Specify if you agree with the following statements about the risks of ACF:**

*2.5.1 ACF leads to an increased risk of false-positive diagnoses of TB.*

| Strongly  agree | Agree | Neither agree nor disagree | Disagree | Strongly disagree |
| --- | --- | --- | --- | --- |

*2.5.2 ACF leads to increased risk of stigma and discrimination.*

| Strongly  agree | Agree | Neither agree nor disagree | Disagree | Strongly disagree |
| --- | --- | --- | --- | --- |

*2.5.3 ACF leads to increased worry about health among households screened.*

| Strongly  agree | Agree | Neither agree nor disagree | Disagree | Strongly disagree |
| --- | --- | --- | --- | --- |

*2.5.4 ACF leads to increased patient cost.*

| Strongly  agree | Agree | Neither agree nor disagree | Disagree | Strongly disagree |
| --- | --- | --- | --- | --- |

*2.5.5 ACF leads to increased health system costs in the short term.*

| Strongly  agree | Agree | Neither agree nor disagree | Disagree | Strongly disagree |
| --- | --- | --- | --- | --- |

*2.5.6 ACF leads to increased health system costs in the long term (over 10 years).*

| Strongly  agree | Agree | Neither agree nor disagree | Disagree | Strongly disagree |
| --- | --- | --- | --- | --- |

**2.6 Are there other risks of ACF you would like to mention? Please elaborate.**

|  |
| --- |

**2.7 Why did you agree/disagree with the risks of ACF? Please elaborate.**

|  |
| --- |

**3 ACF policy in your country**

**3.1 Does a written ACF policy exist in your country (either stand-alone or as part of a national strategic plan)?**

| Yes | No |
| --- | --- |

- If no, continue at question 4.
- If yes, continue with the next question (3.2).

**3.2 Explain the ACF policy in your country by briefly answering the following questions:**

*3.2.1 What year was the ACF policy published?*

|  |
| --- |

*3.2.2 Which risk/priority group(s) are targeted for ACF according to the policy?*

|  |
| --- |

*3.2.3 What screening algorithms are used?*

|  |
| --- |

*3.2.4 Has the ACF policy been evaluated/formally assessed?*

| Yes | No |
| --- | --- |

- If no, continue with question 4.
- If yes, continue with the next question (3.1.5).

*3.2.5 Describe the impact of the ACF policy in your country.*

|  |
| --- |

**4 Use of evidence**

***4.1 Specify how often and when in the ACF policy process different types of evidence were used to inform the ACF policy:***

*4.1.1 How often have* ***WHO guidelines*** *been used in the ACF policy process?*

| Never | Rarely | Sometimes | Often | Always |
| --- | --- | --- | --- | --- |

*4.1.2 When in the ACF policy process have* ***WHO guidelines*** *been used? Please tick all that apply.*

- Agenda-setting
- Policy formulation
- Policy implementation
- Policy evaluation

Please elaborate:

|  |
| --- |

*4.1.3 How often has* ***international scientific evidence*** *(e.g. publications in international journals) been used in the ACF policy process?*

| Never | Rarely | Sometimes | Often | Always |
| --- | --- | --- | --- | --- |

*4.1.4 When in the ACF policy process has* ***international scientific evidence*** *been used? Please tick all that apply.*

- Agenda-setting
- Policy formulation
- Policy implementation
- Policy evaluation

Please elaborate:

|  |
| --- |

*4.1.5 How often has* ***national scientific evidence*** *(e.g. publications in national journals) been used in the ACF policy process?*

| Never | Rarely | Sometimes | Often | Always |
| --- | --- | --- | --- | --- |

*4.1.6 When in the ACF policy process has* ***national scientific evidence*** *been used? Please tick all that apply.*

- Agenda-setting
- Policy formulation
- Policy implementation
- Policy evaluation

Please elaborate:

|  |
| --- |

*4.1.7 How often has* ***expert knowledge*** *been used in the ACF policy process?*

| Never | Rarely | Sometimes | Often | Always |
| --- | --- | --- | --- | --- |

*4.1.8 When in the ACF policy process has* ***expert knowledge*** *been used? Please tick all that apply.*

- Agenda-setting
- Policy formulation
- Policy implementation
- Policy evaluation

Please elaborate:

|  |
| --- |

*4.1.9 How often has* ***personal experience*** *been used in the ACF policy process?*

| Never | Rarely | Sometimes | Often | Always |
| --- | --- | --- | --- | --- |

*4.1.10 When in the ACF policy process has* ***personal experience*** *been used? Please tick all that apply.*

- Agenda-setting
- Policy formulation
- Policy implementation
- Policy evaluation

Please elaborate:

|  |
| --- |

**4.2 Are there other types of evidence that were used to inform the ACF policy? Please elaborate.**

|  |
| --- |

**5 Policy development**

**5.1 Which type of stakeholders were involved in the ACF policy development process? Please tick all that apply.**

- Policy-makers in national government
- Policy-makers in sub-national government
- Managers in a district/region
- Managers in a healthcare institution (e.g. hospital)
- Managers in a non-governmental organization
- International researchers
- National researchers
- Civil society groups
- Professional associations
- International organizations
- Donor agencies
- Pharmaceutical or biotechnology companies
- Citizens
- Patients
- Others (please elaborate)

|  |
| --- |

**5.2 Who do you consider the most powerful stakeholder in developing the ACF policy? Please elaborate.**

|  |
| --- |

**5.3 Specify how much different contextual factors influenced the development of the ACF policy:**

*5.3.1 Factors at the level of the* ***country context*** *(e.g. culture or political system) influenced the development of the ACF policy.*

| Very high degree | High degree | Moderate degree | Small degree | Not at all |
| --- | --- | --- | --- | --- |

Please elaborate:

|  |
| --- |

*5.3.2 Factors at the level of the* ***health system context*** *influenced the development of the ACF policy.*

| Very high degree | High degree | Moderate degree | Small degree | Not at all |
| --- | --- | --- | --- | --- |

Please elaborate:

|  |
| --- |

*5.3.3 Factors at the level of* ***organizations or the community*** *influenced the development of the ACF policy.*

| Very high degree | High degree | Moderate degree | Small degree | Not at all |
| --- | --- | --- | --- | --- |

Please elaborate:

|  |
| --- |

*5.3.4 Factors at the level of* ***funders’ priorities*** *influenced the development of the ACF policy.*

| Very high degree | High degree | Moderate degree | Small degree | Not at all |
| --- | --- | --- | --- | --- |

Please elaborate:

|  |
| --- |

*5.3.5 Factors at the level of the* ***individual*** *influenced the development of the ACF policy.*

| Very high degree | High degree | Moderate degree | Small degree | Not at all |
| --- | --- | --- | --- | --- |

Please elaborate:

|  |
| --- |

*5.3.6 Other factors that influenced the development of the ACF policy:*

|  |
| --- |

**5.4 What do you consider the most powerful influence in developing the ACF policy?**

|  |
| --- |

**6 Policy implementation**

**6.1 Our country’s ACF policy is being implemented.**

| Yes | Partly | No |
| --- | --- | --- |

Please elaborate:

|  |
| --- |

*6.1.1 Describe implementation strategy/strategies:*

|  |
| --- |

**6.2 Which type of stakeholders are involved in the ACF policy implementation process? Please tick all that apply.**

- Policy-makers in national government
- Policy-makers in sub-national government
- Managers in a district/region
- Managers in a healthcare institution (e.g. hospital)
- Managers in a non-governmental organization
- International researchers
- National researchers
- Civil society groups
- Professional associations
- International organizations
- Donor agencies
- Pharmaceutical or biotechnology companies
- Citizens
- Patients
- Others (please elaborate)

|  |
| --- |

**6.3 Who do you consider the most powerful stakeholder in implementing the ACF policy?**

|  |
| --- |

**6.4 Specify how much different contextual factors influenced the implementation of the ACF policy:**

*6.4.1 Factors at the level of the* ***country context*** *(e.g. culture or political system) influenced the implementation of the ACF policy.*

| Very high degree | High degree | Moderate degree | Small degree | Not at all |
| --- | --- | --- | --- | --- |

Please elaborate:

|  |
| --- |

*6.4.2 Factors at the level of the* ***health system context*** *influenced the implementation of the ACF policy.*

| Very high degree | High degree | Moderate degree | Small degree | Not at all |
| --- | --- | --- | --- | --- |

Please elaborate:

|  |
| --- |

*6.4.3 Factors at the level of* ***organizations or the community*** *influenced the implementation of the ACF policy.*

| Very high degree | High degree | Moderate degree | Small degree | Not at all |
| --- | --- | --- | --- | --- |

Please elaborate:

|  |
| --- |

*6.4.4 Factors at the level of* ***funders’ priorities*** *influenced the implementation of the ACF policy.*

| Very high degree | High degree | Moderate degree | Small degree | Not at all |
| --- | --- | --- | --- | --- |

Please elaborate:

|  |
| --- |

*6.4.5 Factors at the level of the* ***individual*** *influenced the implementation of the ACF policy.*

| Very high degree | High degree | Moderate degree | Small degree | Not at all |
| --- | --- | --- | --- | --- |

Please elaborate:

|  |
| --- |

*6.4.6 Other factors that influenced the implementation of the ACF policy:*

|  |
| --- |

**6.5 What do you consider the most powerful influence in implementing the ACF policy?**

|  |
| --- |

**7 Scale-up**

**7.1 In your opinion, should ACF be scaled up in your country?**

| Yes | No |
| --- | --- |

**7.1.1 If no, describe why:**

|  |
| --- |

**7.1.2 If yes, describe the needs for improved implementation and scale-up at the following levels:**

*7.1.2.1 At the level of the* ***country context*** *(e.g. culture or political system)*

|  |
| --- |

*7.1.2.2 At the level of the* ***health system*** *context*

|  |
| --- |

*7.1.2.3 At the level of* ***organizations or the community***

|  |
| --- |

*7.1.2.4 At the level of* ***funders’ priorities***

|  |
| --- |

*7.1.2.5 At the level of the* ***individual***

|  |
| --- |

*7.1.2.6 Other factors:*

|  |
| --- |

**8. Resources**

**8.1 Do sufficient financial resources for ACF exist in our country?**

| Yes | No |
| --- | --- |

**8.1.1 If no, could you describe any strategies for generating financial resources?**

|  |
| --- |

**8.1.2 Describe the following resources:**

*8.1.2.1 Total budget of the National TB Programme (in USD)*

|  |
| --- |

*8.1.2.2 List sources of the National TB Program’s budget (incl. the approximate proportion of the total budget)*

|  | Funding source | Proportion of total budget (in %) |
| --- | --- | --- |
| 1 |  |  |
| 2 |  |  |
| 3 |  |  |
| 4 |  |  |
| 5 |  |  |
| 6 |  |  |

*8.1.2.3 Estimated proportion of the National TB Program’s budget spent on ACF*

|  |
| --- |

*8.1.2.4 Estimated proportion of international donor funding received spent on ACF*

|  |
| --- |

**8.1.3 Does your country receive Global Fund support for TB?**

| Yes | No |
| --- | --- |

*8.1.3.1 If yes, what estimated proportion of the Global Fund budget is for ACF?*

|  |
| --- |

**8.2. Do sufficient human resources for ACF exist in your country?**

| Yes | No |
| --- | --- |

**8.2.1 If no, could you describe any strategies for fighting human resource constraints?**

|  |
| --- |

**8.2.2 Describe the existing human resources:**

|  |
| --- |

**9. Monitoring and evaluation**

**9.1 Does a system for monitoring and evaluation of ACF exists in our country?**

| Yes | No |
| --- | --- |

*9.1.1 If no, could you describe any plans for monitoring and evaluation?*

|  |
| --- |

9.1.2 If yes, describe the most important indicators used.

|  |
| --- |

**10. Concluding questions**

**10.1 How important is ACF for TB prevention and care in your country?**

| Very important | Important | So-so | Slightly important | Not at all important |
| --- | --- | --- | --- | --- |

**10.2 Describe the most important components of successful ACF:**

|  |
| --- |

**10.3 Describe how to make ACF sustainable:**

|  |
| --- |

**10.4 Describe your most important lesson learned related to ACF:**

|  |
| --- |

**10.5 Do you have any additional comments?**

|  |
| --- |

***Thank you very much for participating in this survey.***
