# Supplementary material for: Active case-finding policy development, implementation and scale-up in high-burden countries: A mixed-methods survey with National Tuberculosis Programme managers and document review
Source: PLoS One. 2020 Oct 28;15(10):e0240696. doi: 10.1371/journal.pone.0240696 (PMC7592767; doi:10.1371/journal.pone.0240696)
Supplement: S2 Data — (PDF) [file pone.0240696.s004.pdf]

NTP manager survey

Codebook

Data Dictionary Codebook

13/02/2020 13:40

	#	Variable / Field Name	Field Label<br>*Field Note*	Field Attributes (Field Type, Validation, Choices, Calculations, etc.)
Instrument: **My First Instrument** (my_first_instrument)				
	1	record_id	Record ID	text
	2	s1q1_sex	Section Header: *Section 1: Personal background*<br>1 Sex	radio<br>0 Female<br>1 Male
	3	s1q2_age	2 Age group (years)	radio<br>0 20-29<br>1 30-39<br>2 40-49<br>3 50-59<br>6 60-69<br>7 >70

	4	s1q3_country	3 Country	dropdown, Required<br>0 Angola<br>1 Bangladesh<br>2 Brazil<br>3 Cambodia<br>4 China<br>5 Congo<br>6 Central African Republic<br>7 DPR Korea<br>8 DR Cong<br>9 Ethiopia<br>10 India<br>11 Indonesia<br>12 Kenya<br>13 Lesotho<br>14 Liberia<br>15 Mozambique<br>16 Myanmar<br>17 Namibia<br>18 Nigeria<br>19 Pakistan<br>20 Papua New Guinea<br>21 Philippines<br>22 Russian Federation<br>23 Sierra Leone<br>24 South Africa<br>25 Tanzania<br>26 Thailand<br>27 Vietnam<br>28 Zambia<br>29 Zimbabwe
	5	s1q4_org	4 Organization	text
	6	s1q5_job	5 Job title	text
	7	s1q6_role	6 Role	radio<br>0 Policy-maker<br>1 Researcher<br>2 Other
	8	s1q6_1_expres<br>Show the field ONLY if: [s1q6_role] = '0' or [s1q6_rol e] = '2'	6.1 If you identified yourself as a policy-maker or other stakeholder, please indicate if you have training and/or extensive experience as a researcher	yesno<br>1 Yes<br>0 No
	9	s1q6_2_exppol<br>Show the field ONLY if: [s1q6_role] = '1'	6.2 If you identified yourself as a researcher, please indicate if you have training and/or extensive experience as a policy-maker.	yesno<br>1 Yes<br>0 No
	10	s1q7_startjob	7 What year did you start working in this job?	text (number, Min: 1940, Max: 2019)
	11	s1q8_starttb	8 What year did you start working in the area of TB?	text (number, Min: 1940, Max: 2019)

	12	s2q1_1_benearly	Section Header: *Section 2: ACF policies - general view*<br>1.1 ACF leads to early detection, diagnosis and treatment.	radio (Matrix)<br>1 Strongly agree<br>2 Agree<br>3 Neither agree nor disagree<br>4 Disagree<br>5 Strongly disagree
	13	s2q1_2_bentrans	1.2 ACF leads to reduced transmission and incidence of TB.	radio (Matrix)<br>1 Strongly agree<br>2 Agree<br>3 Neither agree nor disagree<br>4 Disagree<br>5 Strongly disagree
	14	s2q1_3_bentreat	1.3 ACF leads to improved treatment outcomes.	radio (Matrix)<br>1 Strongly agree<br>2 Agree<br>3 Neither agree nor disagree<br>4 Disagree<br>5 Strongly disagree
	15	s2q1_4_benhs	1.4 ACF leads to reduced future health system cost.	radio (Matrix)<br>1 Strongly agree<br>2 Agree<br>3 Neither agree nor disagree<br>4 Disagree<br>5 Strongly disagree
	16	s2q1_5_beneco	1.5 ACF has positive social and economic consequences for the TB patient.	radio (Matrix)<br>1 Strongly agree<br>2 Agree<br>3 Neither agree nor disagree<br>4 Disagree<br>5 Strongly disagree
	17	s2q2_benother	2 Are there other benefits of ACF you would like to mention? Please elaborate.	text<br>Custom alignment: LV
	18	s2q3_benwhy	3 Why did you agree/disagree with the benefits of ACF? Please elaborate.	text<br>Custom alignment: LV
	19	s2q4_rank	4 How would you rank the importance of ACF among other TB interventions for early case detection?<br>*Please rank from 1-7 (1 = most important)*	checkbox<br>0 s2q4_rank___0 Implementing ACF<br>1 s2q4_rank___1 Improving knowledge about TB among patients and the community<br>2 s2q4_rank___2 Reducing access barriers to health care<br>3 s2q4_rank___3 Reducing stigma<br>4 s2q4_rank___4 Implementing proven diagnostic tools<br>5 s2q4_rank___5 Training health workers<br>6 s2q4_rank___6 Other<br>Custom alignment: LV
	20	s2q4a_impacf<br>Show the field ONLY if:<br>[s2q4_rank(0)] = '1'	4a Implementing ACF	text (number, Min: 1, Max: 7)<br>Custom alignment: LV

	21	s2q4b_imprknowledge<br><br>Show the field ONLY if:<br>[s2q4_rank(1)] = '1'	4b Improving knowledge about TB among patients and the community	text (number, Min: 1, Max: 7)<br>Custom alignment: LV
	22	s2q4c_redaccess<br><br>Show the field ONLY if:<br>[s2q4_rank(2)] = '1'	4c Reducing access barriers	text (number, Min: 1, Max: 7)<br>Custom alignment: LV
	23	s2q4d_redstigma<br><br>Show the field ONLY if:<br>[s2q4_rank(3)] = '1'	4d Reducing stigma	text (number, Min: 1, Max: 7)<br>Custom alignment: LV
	24	s2q4e_impproven<br><br>Show the field ONLY if:<br>[s2q4_rank(4)] = '1'	4e Implementing proven diagnostic tools	text (number, Min: 1, Max: 7)<br>Custom alignment: LV
	25	s2q4f_train<br><br>Show the field ONLY if:<br>[s2q4_rank(6)] = '1'	4f Training health workers	text (number, Min: 1, Max: 7)<br>Custom alignment: LV
	26	s2q4g_other<br><br>Show the field ONLY if:<br>[s2q4_rank(6)] = '1'	4g Other (please specify)	text (number, Min: 1, Max: 7)<br>Custom alignment: LV
	27	s2q4_1_rankwhy	4.1 Please elaborate on why you ranked the TB interventions for early case detection as you did.	text<br>Custom alignment: LV
	28	s2q4_2_nsp	4.2 ACF contributes to the goals of your National Strategic Plan.	radio (Matrix)<br>1 Strongly agree<br>2 Agree<br>3 Neither agree nor disagree<br>4 Disagree<br>5 Strongly disagree
	29	s2q4_2a_ela<br><br>Show the field ONLY if:<br>[s2q4_2_nsp] = '1' or [s2q4_2_nsp] = '2' or [s2q4_2_nsp] = '3' or [s2q4_2_nsp] = '4' or [s2q4_2_nsp] = '5'	4.2a Please elaborate	text<br>Custom alignment: LV
	30	s2q5_1_riskfalspos	5.1 ACF leads to an increased risk of false-positive diagnoses of TB.	radio (Matrix)<br>1 Strongly agree<br>2 Agree<br>3 Neither agree nor disagree<br>4 Disagree<br>5 Strongly disagree
	31	s2q5_2riskstig	5.2 ACF leads to increased risk of stigma and discrimination.	radio (Matrix)<br>1 Strongly agree<br>2 Agree<br>3 Neither agree nor disagree<br>4 Disagree<br>5 Strongly disagree
	32	s2q5_3_riskworry	5.3 ACF leads to increased worry about health among households screened.	radio (Matrix)<br>1 Strongly agree<br>2 Agree<br>3 Neither agree nor disagree<br>4 Disagree<br>5 Strongly disagree

	33	s2q5_4_riskpatcost	5.4 ACF leads to increased patient cost.	radio (Matrix)<br>1 Strongly agree<br>2 Agree<br>3 Neither agree nor disagree<br>4 Disagree<br>5 Strongly disagree
	34	s2q5_5_riskhscostshort	5.5 ACF leads to increased health system costs in the short term.	radio (Matrix)<br>1 Strongly agree<br>2 Agree<br>3 Neither agree nor disagree<br>4 Disagree<br>5 Strongly disagree
	35	s2q5_6_riskhscostlong	5.6 ACF leads to increased health system costs in the long term (over 10 years).	radio (Matrix)<br>1 Strongly agree<br>2 Agree<br>3 Neither agree nor disagree<br>4 Disagree<br>5 Strongly disagree
	36	s2q6_otherrisks	6 Are there other risks of ACF you would like to mention?	text<br>Custom alignment: LV
	37	s1q7_whyagr	7 Why did you agree/disagree with the risks of ACF? Please elaborate.	text<br>Custom alignment: LV
	38	s3q1_natlacfpol	Section Header: *Section 3: ACF policy in your country*<br>1 Does a written ACF policy exist in your country (either stand-alone or as part of a national strategic plan)?	yesno<br>1 Yes<br>0 No<br>Custom alignment: LV
	39	s3q2_1_year<br>Show the field ONLY if:<br>[s3q1_natlacfpol] = '1'	2.1 What year was the ACF policy published?	text<br>Custom alignment: LV
	40	s3q2_2_riskgrp<br>Show the field ONLY if:<br>[s3q1_natlacfpol] = '1'	2.2 Which risk/priority group(s) are targeted for ACF according to the policy?	text<br>Custom alignment: LV
	41	s3q2_3_algor<br>Show the field ONLY if:<br>[s3q1_natlacfpol] = '1'	2.3 What screening algorithms are used?	text<br>Custom alignment: LV
	42	s3q2_4_eval<br>Show the field ONLY if:<br>[s3q1_natlacfpol] = '1'	2.4 Has the ACF policy been evaluated/formally assessed?	yesno<br>1 Yes<br>0 No<br>Custom alignment: LV
	43	s3q2_5_impact<br>Show the field ONLY if:<br>[s3q2_4_eval] = '1'	2.5 Describe the impact of the ACF policy in your country.	text<br>Custom alignment: LV
	44	s4q1_1_whoguide	Section Header: *Section 4: Use of evidence*<br>1.1 How often have WHO guidelines been used in the ACF policy process?	radio (Matrix)<br>1 Never<br>2 Rarely<br>3 Sometimes<br>4 Often<br>5 Always

	45	s4q1_2_when<br>Show the field ONLY if:<br>[s4q1_1_whoguide] = '2' or [s4q1_1_whoguide] = '3' or [s4q1_1_whoguide] = '4' or [s4q1_1_whoguide] = '5'	1.2 When in the ACF policy process have WHO guidelines been used? Please tick all that apply.	checkbox<br>0 s4q1_2_when___0 Agenda-setting<br>1 s4q1_2_when___1 Policy formulation<br>2 s4q1_2_when___2 Policy implementation<br>3 s4q1_2_when___3 Policy evaluation<br>Custom alignment: LV
	46	s4q1_2a_ela	1.2a Please elaborate	text<br>Custom alignment: LV
	47	s4q1_3_intlsci	1.3 How often has international scientific evidence (e.g. publications in international journals) been used in the ACF policy process?	radio (Matrix)<br>1 Never<br>2 Rarely<br>3 Sometimes<br>4 Often<br>5 Always
	48	s4q1_4_when<br>Show the field ONLY if:<br>[s4q1_3_intlsci] = '2' or [s4q1_3_intlsci] = '3' or [s4q1_3_intlsci] = '4' or [s4q1_3_intlsci] = '5'	1.4 When in the ACF policy process has international scientific evidence been used? Please tick all that apply.	checkbox<br>0 s4q1_4_when___0 Agenda-setting<br>1 s4q1_4_when___1 Policy formulation<br>2 s4q1_4_when___2 Policy implementation<br>3 s4q1_4_when___3 Policy evaluation<br>Custom alignment: LV
	49	s4q1_4a_ela	1.4a Please elaborate	text<br>Custom alignment: LV
	50	s4q1_5_natlsci	1.5 How often has national scientific evidence (e.g. publications in national journals) been used in the ACF policy process?	radio (Matrix)<br>1 Never<br>2 Rarely<br>3 Sometimes<br>4 Often<br>5 Always
	51	s4q1_6_when<br>Show the field ONLY if:<br>[s4q1_5_natlsci] = '2' or [s4q1_5_natlsci] = '3' or [s4q1_5_natlsci] = '4' or [s4q1_5_natlsci] = '5'	1.6 When in the ACF policy process has national scientific evidence been used? Please tick all that apply.	checkbox<br>0 s4q1_6_when___0 Agenda-setting<br>1 s4q1_6_when___1 Policy formulation<br>2 s4q1_6_when___2 Policy implementation<br>3 s4q1_6_when___3 Policy evaluation<br>Custom alignment: LV
	52	s4q1_6a_ela	1.6a Please elaborate	text<br>Custom alignment: LV
	53	s4q1_7_expknow	1.7 How often has expert knowledge been used in the ACF policy process?	radio (Matrix)<br>1 Never<br>2 Rarely<br>3 Sometimes<br>4 Often<br>5 Always
	54	s4q1_8_when<br>Show the field ONLY if:<br>[s4q1_7_expknow] = '2' or [s4q1_7_expknow] = '3' or [s4q1_7_expknow] = '4' or [s4q1_7_expknow] = '5'	1.8 When in the ACF policy process has expert knowledge been used? Please tick all that apply.	checkbox<br>0 s4q1_8_when___0 Agenda-setting<br>1 s4q1_8_when___1 Policy formulation<br>2 s4q1_8_when___2 Policy implementation<br>3 s4q1_8_when___3 Policy evaluation<br>Custom alignment: LV

	55	s4q1_8a_ela	1.8a Please elaborate	text<br>Custom alignment: LV
	56	s4q1_9_persexp	1.9 How often has personal experience been used in the ACF policy process?	radio (Matrix)<br>1 Never<br>2 Rarely<br>3 Sometimes<br>4 Often<br>5 Always
	57	s4q1_10_when<br>Show the field ONLY if:<br>[s4q1_9_persexp] = '2' or [s4q1_9_persexp] = '3' or [s4q1_9_persexp] = '4' or [s4q1_9_persexp] = '5'	1.10 When in the ACF policy process has personal experience been used? Please tick all that apply.	checkbox<br>0 s4q1_10_when___0 Agenda-setting<br>1 s4q1_10_when___1 Policy formulation<br>2 s4q1_10_when___2 Policy implementation<br>3 s4q1_10_when___3 Policy evaluation<br>Custom alignment: LV
	58	s4q1_10a_ela	1.10a Please elaborate	text<br>Custom alignment: LV
	59	s4q2_other	2 Are there other types of evidence that were used to inform the ACF policy? Please elaborate.	text<br>Custom alignment: LV
	60	s5q1_stakehtype	Section Header: *Section 5: Policy development*<br>1 Which type of stakeholders were involved in the ACF policy development process? Please tick all that apply.	checkbox<br>0 s5q1_stakehtype___0 Policy-makers in national government<br>1 s5q1_stakehtype___1 Policy-makers in sub-national government<br>2 s5q1_stakehtype___2 Managers in a district/region<br>3 s5q1_stakehtype___3 Managers in a healthcare institution (e.g. hospital)<br>4 s5q1_stakehtype___4 Managers in a non-governmental organization<br>5 s5q1_stakehtype___5 International researchers<br>6 s5q1_stakehtype___6 National researchers<br>7 s5q1_stakehtype___7 Civil society groups<br>8 s5q1_stakehtype___8 Professional associations<br>9 s5q1_stakehtype___9 International organizations<br>10 s5q1_stakehtype___10 Donor agencies<br>11 s5q1_stakehtype___11 Pharmaceutical or biotechnology companies<br>12 s5q1_stakehtype___12 Citizens<br>13 s5q1_stakehtype___13 Patients<br>14 s5q1_stakehtype___14 Others<br>Custom alignment: LV
	61	s5q1a_ela	1a Please elaborate	text<br>Custom alignment: LV
	62	s5q2_power	2 Who do you consider the most powerful stakeholder in developing the ACF policy? Please elaborate.	text<br>Custom alignment: LV

	63	s5q3_1_infdevcountry	3.1 Factors at the level of the country context (e.g. culture or political system) influenced the development of the ACF policy.	radio (Matrix)<br>1 Very high degree<br>2 High degree<br>3 Moderate degree<br>4 Small degree<br>5 Not at all
	64	s5q3_1a_ela	3.1a Please elaborate	text<br>Custom alignment: LV
	65	s5q3_2_infdevhs	3.2 Factors at the level of the health system context influenced the development of the ACF policy.	radio (Matrix)<br>1 Very high degree<br>2 High degree<br>3 Moderate degree<br>4 Small degree<br>5 Not at all
	66	s5q3_2a_ela	3.2a Please elaborate	text<br>Custom alignment: LV
	67	s5q3_3_infdevorg	3.3 Factors at the level of organizations or the community influenced the development of the ACF policy.	radio (Matrix)<br>1 Very high degree<br>2 High degree<br>3 Moderate degree<br>4 Small degree<br>5 Not at all
	68	s5q3_3a_ela	3.3a Please elaborate	text<br>Custom alignment: LV
	69	s5q3_4_infdevfund	3.4 Factors at the level of funders' priorities influenced the development of the ACF policy.	radio (Matrix)<br>1 Very high degree<br>2 High degree<br>3 Moderate degree<br>4 Small degree<br>5 Not at all
	70	s5q3_4a_ela	3.4a Please elaborate	text<br>Custom alignment: LV
	71	s5q3_5_infdevind	3.5 Factors at the level of the individual influenced the development of the ACF policy.	radio (Matrix)<br>1 Very high degree<br>2 High degree<br>3 Moderate degree<br>4 Small degree<br>5 Not at all
	72	s5q3_5a_ela	3.5a Please elaborate	text
	73	s5q3_6_other	3.6 Other factors that influenced the development of the ACF policy	text
	74	s5q4_power	4 What do you consider the most powerful influence in developing the ACF policy?	text
	75	s6q1_acfimp	Section Header: *Section 6: Policy implementation*<br>1 Our country's ACF policy is being implemented	radio (Matrix)<br>1 Yes<br>2 Partly<br>3 No
	76	s6q1a_ela	1a Please elaborate	text
	77	s6q1_1_impstrat	1.1 Describe implementation strategy/strategies	text

	78	s6q2_stakehtype	2 Which type of stakeholders are involved in the ACF policy implementation process? Please tick all that apply.	checkbox<br>0 s6q2_stakehtype___0 Policy-makers in national government<br>1 s6q2_stakehtype___1 Policy-makers in sub-national government<br>2 s6q2_stakehtype___2 Managers in a district/region<br>3 s6q2_stakehtype___3 Managers in a healthcare institution (e.g. hospital)<br>4 s6q2_stakehtype___4 Managers in a non-governmental organization<br>5 s6q2_stakehtype___5 International researchers<br>6 s6q2_stakehtype___6 National researchers<br>7 s6q2_stakehtype___7 Civil society groups<br>8 s6q2_stakehtype___8 Professional associations<br>9 s6q2_stakehtype___9 International organizations<br>10 s6q2_stakehtype___10 Donor agencies<br>11 s6q2_stakehtype___11 Pharmaceutical or biotechnology companies<br>12 s6q2_stakehtype___12 Citizens<br>13 s6q2_stakehtype___13 Patients<br>14 s6q2_stakehtype___14 Others
	79	s6q2a_ela	2a Please elaborate	text
	80	s6q3_power	3 Who do you consider the most powerful stakeholder in implementing the ACF policy?	text
	81	s6q4_1_infimpcountry	4.1 Factors at the level of the country context (e.g. culture or political system) influenced the implementation of the ACF policy.	radio (Matrix)<br>1 Very high degree<br>2 High degree<br>3 Moderate degree<br>4 Small degree<br>5 Not at all
	82	s6q4_1a_ela	4.1a Please elaborate	text
	83	s6q4_2_infimphs	4.2 Factors at the level of the health system context influenced the implementation of the ACF policy.	radio (Matrix)<br>1 Very high degree<br>2 High degree<br>3 Moderate degree<br>4 Small degree<br>5 Not at all
	84	s6q4_2a_ela	4.2a Please elaborate	text
	85	s6q4_3_infimporg	4.3 Factors at the level of organizations or the community influenced the implementation of the ACF policy.	radio (Matrix)<br>1 Very high degree<br>2 High degree<br>3 Moderate degree<br>4 Small degree<br>5 Not at all
	86	s6q4_3a_ela	4.3a Please elaborate	text

	87	s6q4_4_infimpfund	4.4 Factors at the level of funders' priorities influenced the implementation of the ACF policy.	radio (Matrix)<br>1 Very high degree<br>2 High degree<br>3 Moderate degree<br>4 Small degree<br>5 Not at all
	88	s6q4_4a_ela	4.4a Please elaborate	text
	89	s6q4_5_infimpind	4.5 Factors at the level of the individual influenced the implementation of the ACF policy.	radio (Matrix)<br>1 Very high degree<br>2 High degree<br>3 Moderate degree<br>4 Small degree<br>5 Not at all
	90	s6q4_5a_ela	4.5a Please elaborate	text
	91	s6q4_6_other	4.6 Other factors that influenced the implementation of the ACF policy:	text
	92	s6q5_power	5 What do you consider the most powerful influence in implementing the ACF policy?	text
	93	s7q1_scaleup	Section Header: *Section 7: Scale-up*<br>1 In your opinion, should ACF be scaled up in your country?	yesno<br>1 Yes<br>0 No
	94	s7q1_1_whynot<br>Show the field ONLY if:<br>[s7q1_scaleup] = '0'	1.1 Describe why not:	text
	95	s7q2_1_needscountry<br>Show the field ONLY if:<br>[s7q1_scaleup] = '1'	2.1 Describe the needs for improved implementation and scale-up at the level of the country context (e.g. culture or political system).	text
	96	s7q2_2_needshs<br>Show the field ONLY if:<br>[s7q1_scaleup] = '1'	2.2 Describe the needs for improved implementation and scale-up at the level of the health system context.	text
	97	s7q2_3_needsorg<br>Show the field ONLY if:<br>[s7q1_scaleup] = '1'	2.3 Describe the needs for improved implementation and scale-up at the level of organizations or the community.	text
	98	s7q2_4_needsfund<br>Show the field ONLY if:<br>[s7q1_scaleup] = '1'	2.4 Describe the needs for improved implementation and scale-up at the level of funders' priorities.	text
	99	s7q2_5_needsind<br>Show the field ONLY if:<br>[s7q1_scaleup] = '1'	2.5 Describe the needs for improved implementation and scale-up at the level of the individual.	text
	100	s7q2_6_needsother<br>Show the field ONLY if:<br>[s7q1_scaleup] = '1'	2.6 Are there any other needs for improved implementation and scale-up?	text
	101	s8q1_finres	Section Header: *Section 8: Resources*<br>1 Do sufficient financial resources for ACF exist in our country?	yesno<br>1 Yes<br>0 No
	102	s8q1_1_stratfinres<br>Show the field ONLY if:<br>[s8q1_finres] = '0'	1.1 Could you describe any strategies for generating financial resources?	text
	103	s8q2_ntpbudget	2 Describe the total budget of the National TB Programme (in USD).	text (number)
	104	s8q3_1_proportiondom	3.1 Proportion of domestic funding of total budget (in %)	text (number, Min: 1, Max: 100)

	105	s8q3_2_proportiongf	3.2 Proportion of Global Fund funding of total budget (in %)	text (number, Min: 1, Max: 100)
	106	s8q3_3_proportionusaid	3.3 Proportion of USAID funding of total budget (in %)	text (number, Min: 1, Max: 100)
	107	s8q3_4_proportionwb	3.4 Proportion of World Bank funding of total budget (in %)	text (number, Min: 1, Max: 100)
	108	s8q3_5_proportioncdc	3.5 Proportion of CDC funding of total budget (in %)	text (number, Min: 1, Max: 100)
	109	s8q3_6_proportionausaid	3.6 Proportion of AUSAID funding of total budget (in %)	text (number, Min: 1, Max: 100)
	110	s8q3_7_proportionmsf	3.7 Proportion of MSF funding of total budget (in %)	text (number, Min: 1, Max: 100)
	111	s8q3_8_proportionwho	3.8 Proportion of WHO funding of total budget (in %)	text (number, Min: 1, Max: 100)
	112	s8q3_9_proportionkncv	3.9 Proportion of KNCV funding of total budget (in %)	text (number, Min: 1, Max: 100)
	113	s8q3_10_proportionstp	3.10 Proportion of Stop TB Partnership/TB Reach funding of total budget (in %)	text (number, Min: 1, Max: 100)
	114	s8q3_11_proportionother	3.11 Proportion of other funding of total budget (in %)	text (number, Min: 1, Max: 100)
	115	s8q3_12_comment	3.12 Comment	text
	116	s8q4_ntpbudgacf	4 Estimated proportion of the National TB Program's budget spent on ACF	text (number, Min: 1, Max: 100)
	117	s8q5_donorbudgacf	5 Estimated proportion of international donor funding received spent on ACF	text (number, Min: 1, Max: 100)
	118	s8q6_gfsupport	6 Does your country receive Global Fund support for TB?	yesno<br>1 Yes<br>0 No
	119	s8q6_1_gfbudgdacf<br>Show the field ONLY if: [s8q6_gfsupport] = '1'	6.1 What estimated proportion of the Global Fund budget is for ACF?	text (number, Min: 1, Max: 100)
	120	s8q7_hracf	7. Do sufficient human resources for ACF exist in your country?	yesno<br>1 Yes<br>0 No
	121	s8q7_1_hrstrat<br>Show the field ONLY if: [s8q7_hracf] = '0'	7.1 Could you describe any strategies for fighting human resource constraints?	text
	122	s8q8_existhr	8. Describe the existing human resources:	text
	123	s9q1_m_e	Section Header: *Section 9: Monitoring and evaluation*<br>1 Does a system for monitoring and evaluation of ACF exists in our country?	yesno<br>1 Yes<br>0 No
	124	s9q1_1_m_e_plans<br>Show the field ONLY if: [s9q1_m_e] = '0'	1.1 Could you describe any plans for monitoring and evaluation?	text
	125	s9q1_2_m_e_indic<br>Show the field ONLY if: [s9q1_m_e] = '1'	1.2 Describe the most important indicators used.	text
	126	s10q1_acfimp	Section Header: *Section 10: Concluding questions*<br>1 How important is ACF for TB prevention and care in your country?	radio (Matrix)<br>1 Very important<br>2 Important<br>3 So-so<br>4 Slightly important<br>5 Not at all important
	127	s10q2_acfcomp	2 Describe the most important components of successful ACF:	text
	128	s10q3_acfsust	3 Describe how to make ACF sustainable:	text
	129	s10q4_acflesson	4 Describe your most important lesson learned related to ACF:	text
	130	s10q5_acfcomment	5 Do you have any additional comments?	text

	131	my_first_instrument_complete	Section Header: *Form Status*<br>Complete?	dropdown<br>0 Incomplete<br>1 Unverified<br>2 Complete
